# A Professional Development Program That Combines Direct with Indirect Promotion of Self-Regulated Learning for Secondary School Teachers

**DOI:** 10.3390/bs15111512

**Published:** 2025-11-07

**Authors:** Stella Vosniadou, Helen Stephenson, Michael J. Lawson, David Jeffries

**Affiliations:** 1 College of Education, Psychology and Social Work, Flinders University, Adelaide, SA 5042, Australia; mike.lawson@flinders.edu.au; 2 Education Futures, University of South Australia, Adelaide, SA 5000, Australia; helen.stephenson@unisa.edu.au; 3 Australian Council for Educational Research, Melbourne, VIC 3124, Australia; david.jeffries@acer.org

**Keywords:** SRL professional development program, SRL promotion, ICAP, secondary school teachers

## Abstract

A professional development program (PDP) combining direct and indirect promotion of self-regulated learning (SRL) was conducted with secondary school teachers in two parts. In the first part, the teachers were encouraged to promote student cognitive engagement through the inclusion of more interactive and constructive compared to passive and active lesson tasks in their teaching. In the second part, the teachers were provided with information which emphasized the importance of the direct promotion of SRL knowledge and strategies. The teachers were provided with excerpts from videos of classroom instruction to analyze and reflect upon. The results were based on an analysis of the talk and action of the teachers from videoed observations of their own classrooms before the PDP (Round 1), after the first part (Round 2), and after the second part (Round 3). The PDP influenced the teachers’ indirect promotion of SRL through the inclusion of more interactive and constructive and fewer passive and active lesson tasks in their teaching. Direct SRL promotion was also influenced although to a lesser extent, through the teachers’ inclusion of more motivational, metacognitive support statements to students to encourage them to keep on trying, as well as more explicit strategy promotion and reference to the benefits of SRL strategies.

## 1. Introduction

The research presented in this paper investigated the effectiveness of a professional development program (PDP), which combined direct with indirect promotion of self-regulated learning (SRL), and which was mainly addressed to secondary school teachers.

SRL research investigates the cognitive, metacognitive, motivational, and affective knowledge and skills necessary to engage in effective learning. The ability to manage and control one’s learning has become an important requirement for navigating today’s complex and digitized world (Bjork et al., 2013). Educational research has shown that interventions that improve students’ self-regulation have a beneficial impact on student learning and achievement (Dignath et al., 2008; Dignath & Büttner, 2008; Donker et al., 2014; Hattie et al., 1996; Sitzmann & Ely, 2011).

Although there is wide agreement on the benefits of SRL, many students lack the required knowledge and skills of a self-regulated learner (Askell-Williams & Lawson, 2015; Askell-Williams et al., 2012; Cleary & Zimmerman, 2004; Elen & Lowyck, 1999; Schneider et al., 2017; Winne, 2014). Teachers are instrumental in guiding students towards developing SRL by teaching appropriate learning strategies and by designing a learning environment that encourages student independence and cognitive engagement (Azevedo et al., 2008; Hattie, 2011; Paris & Paris, 2001; Perry, 1998; Perry & Rahim, 2011; Veenman, 2017). However, research shows that many teachers may not know enough about SRL and its promotion to help their students become self-regulated learners; Dignath & Büttner, 2018; Hamman et al., 2000; Moely et al., 1992; Spruce & Bol, 2015; Vosniadou et al., 2024).

Responding to the need for better promotion of SRL in schools and recognizing the important role teachers play in this process, various SRL professional development programs (PDPs) have been developed. In some of these programs instructional resources are provided to teachers to support the integration of strategy training within their classroom (e.g., Askell-Williams et al., 2012; Perels et al., 2009), while in other teachers receive professional development on how to promote SRL in classroom teaching (Kohen & Kramarski, 2017; Kramarski, 2017; Neuman & Roskos, 1997; Perry, 1998; Turner, 1995; Xu & Corno, 1998). The present research designed and investigated the effectiveness of a PDP for secondary school teachers’ promotion of SRL in the classroom. The main innovation of the present PDP are as follows: (1) it addressed both the direct and the indirect promotion of SRL; (2) it evaluated the effects of the program on teachers’ actual practices. In the pages that follow, we first review SRL PDP studies focusing on secondary education and then describe the current research.

### 1.1. SRL Professional Development Studies

There has been a proliferation of SRL PDP studies in recent years as researchers and practitioners in education have come to understand the lack of effective SRL instruction. Many programs have been addressed to preservice teachers or to practicing teachers at the kindergarten and primary school levels and relatively few to secondary school teachers. Most of the studies have reported positive results on teachers’ knowledge and beliefs about SRL, on their self-efficacy, on their monitoring skills, or on their self-assessed practices, but few have investigated the impact of professional development on teachers’ actual practices.

Existing PDP programs differ in their duration, in the methods employed, and in the teacher competencies investigated. For example, Perry et al. (2007) implemented a 4-year program in which they grouped 18 preservice teachers into cohorts to cultivate strategies that foster SRL in K-5 classrooms. At the end of the program, the teachers were able to design challenging and meaningful activities that captured students’ interest and involvement. Allshouse (2016), on the other hand, implemented a three-hour PD workshop, which included strategies for analyzing case studies, engaging in self-reflection, and participating in group-based tasks. The results showed statistically significant shifts in understanding and use of SRL when measured before and after training among nine secondary school educators.

Several programs (e.g., Cleary et al., 2022; Kramarski & Gutman, 2006; Peters-Burton & Botov, 2016) employed a multi-day or multi-week format in which teachers met for several hours on different days. Cleary et al. (2022) designed a weeklong thirty-two-hour program for high school science teachers, while Kramarski and Gutman (2006) investigated the impact of a month-long PD program for 64 Israeli elementary school teachers of mathematics. Peters-Burton and Botov (2016) designed a 15-week PD program, with weekly three-hour sessions, aimed at improving science instruction for K-5 teachers. The teachers were involved in collaborative inquiry-oriented lesson analysis, were exposed to exemplars, and created their own lesson plans. The results showed that teachers’ goal setting and monitoring skills developed over time.

Peeters et al. (2014) advocated for a whole-school strategy where all staff fostered conditions for students’ SRL. Yet Heirweg et al. (2021) found that after one year, this approach had a minimal effect on teachers’ confidence in supporting SRL or on student performance. This study involved 10 experimental and 10 control schools and included 40 teachers and 747 students. The program failed to produce notable changes in educators’ beliefs about SRL, self-efficacy, or SRL-promoting practices. Similarly, no significant effects were observed in students’ SRL competencies or academic achievement. These results highlighted the challenges of effectively implementing SRL professional development programs and suggested that more targeted or intensive interventions may be needed to yield significant impacts. 

Crucial for the success of an intervention is the PD and SRL frameworks adopted. Some of the most successful programs have used PD frameworks developed by Darling-Hammond (2016) and Desimone (2009), which emphasize content knowledge, active learning, collaboration, use of models and modeling, access to mentorship, specialist guidance, and chances for constructive feedback and reflection. With respect to SRL, many studies have used Zimmerman’s (2000, 2008) Cyclical Model, which distinguishes a Forethought Phase (planning and goal setting), a Performance Phase (self-monitoring and strategy use), and a Self-Reflection Phase (self-evaluation and adaptive inferences). Others have used other SRL approaches, such as those of Pintrich (2000), Boekaerts and Cascallar (2006), and Efklides (2011), which emphasize contextual and motivational factors and interactions between phases, in addition to SRL competencies. 

Cleary et al. (2022) used the Zimmerman Cyclical Model in the design of a weeklong thirty-two-hour SRL PD workshop which enhanced math teachers’ understanding, confidence, grasp of SRL principles, and practical implementation abilities. The workshop consisted of 16 h of definitions, practical examples, and visual representations of fundamental SRL ideas and methods with didactic reflective activities and another 16 h of SRL expert-structured guided practice opportunities in science areas. Teachers worked collaboratively to consider how to best support students’ SRL processes. The results showed significant improvements in the teachers’ mathematical understanding, increased knowledge and self-efficacy in applying SRL strategies, and positive shifts in attitudes toward SRL implementation. 

Peters-Burton and Botov (2016) used the Zimmerman framework to develop a microanalytic method designed to obtain detailed information about the self-regulatory cycles experienced by 14 high school teachers engaged in inquiry learning. Findings indicated that microanalysis prompts encouraged students to use effective learning techniques, evaluate their approaches, and strengthen their planning and metacognitive skills. They also revealed underlying beliefs and maladaptive processes and helped the instructors to correct them.

Kramarski and Heaysman (2021) developed the Zimmerman (2008) Cyclical Model of SRL into a spiraled three-step professional development program—the Triple SRL–SRT Framework—which highlighted teachers’ multifaceted role as both learners and facilitators of SRL. The program was designed to enhance teachers’ own SRL competencies, integrate SRL into teaching practices, and improve student outcomes through iterative learning cycles, reflective practices, and real-world applications. Other studies (e.g., Galdames-Calderón et al., 2024; Hirt et al., 2025; Karlen et al., 2020) also emphasized the need to unify educators’ abilities as self-directed learners and facilitators of SRL through a comprehensive approach. 

Some intervention research has emphasized metacognitive instruction and reflective activities to enhance SRL among teachers and students (Arvatz & Dori, 2024; Arvatz et al., 2025; Geduld et al., 2024; Kramarski & Gutman, 2006; Phillips et al., 2016). Kramarski and Gutman (2006) investigated the impact of a PD program incorporating the IMPROVE metacognitive self-questioning method on 64 mathematics teachers’ professional knowledge and SRL practices. A quasi-experimental design was used with two groups: one receiving SRL support and one without. The SRL group participated in a month-long PD program integrating the IMPROVE method, focusing on metacognitive questioning techniques. Pre- and post-tests were used to assess teachers’ mathematical and pedagogical knowledge. Observation checklists were used to evaluate the implementation of SRL strategies in teaching practices and self-reflection logs to help teachers document their experiences and reflections on incorporating SRL strategies. The results showed that the teachers in the SRL-supported group showed significant improvements in both mathematical understanding and pedagogical approaches.

Phillips et al. (2016) evaluated the impact of the MAST system, a metacognitive training program for teachers, on secondary students’ math achievement. The participants were 500 students (ages 14–17) and 30 teachers across ten schools. Standardized math tests and teacher SRL knowledge surveys showed significant improvements in student math achievement and teacher SRL knowledge. The authors concluded that metacognitive training for teachers can sustain student achievement gains by fostering SRL. Geduld et al. (2024) examined a metacognitive-based professional development approach to SRL in secondary education with 90 teachers and approximately 900 students (ages 14–18), using teacher reflective journals and SRL observation checklists. Teachers’ reported SRL support increased significantly, while student SRL skills improved moderately. The results support the enhancement of teachers’ metacognitive awareness as a means of developing their ability to foster SRL among students. 

Modeling, authentic simulations, and video viewing have also been used by some researchers to develop teachers’ professional vision related to SRL. Kramarski and Heaysman (2021, 2023) suggested that reviewing classroom video footage enables teachers to observe and imitate SRL behaviors, effectively translating theory into practice. Horlenko et al. (2024) utilized video intervention studies and mobile eye-tracking data to analyze how teachers perceive and respond to students’ SRL behaviors. The findings suggest that video-based interventions can enhance teachers’ ability to notice and interpret SRL-related cues in the classroom and by extension, their instructional practices. Utilizing video recordings of classroom instruction, Rosenthal et al. (2024) discovered that educators tend to promote SRL in subtle ways, primarily emphasizing metacognitive techniques. In contrast Horlenko et al. (2024) employed video-based interventions alongside student feedback, teacher evaluations, and eye-tracking technology to examine how teachers interpret and react to students’ SRL actions.

In summary, the results of the professional development studies conducted so far highlighted several factors that are important to consider in SRL PDPs. Some of these factors are general to all professional development programs, such as the opportunities they provide for active learning, collaboration, modeling, and expert support (Darling-Hammond et al., 2017; Desimone, 2009). Others have to do with the SRL approach adopted and the way this approach was communicated to teachers to enrich their SRL knowledge and promotion practices. A limitation of the research so far is the heterogeneity of these studies. Differences in the methods employed, in the competencies they investigated, and in their participants make it difficult to draw definite conclusions. Most importantly, as mentioned earlier, almost all these programs assessed changes in teachers’ SRL knowledge, beliefs, attitudes, self-efficacy and self-reported SRL practices but much less frequently investigated teachers’ actual practices through observations of their classroom teaching.

Another key shortcoming in current research is its narrow emphasis on direct SRL instruction, often overlooking how SRL can be indirectly nurtured and embedded within content learning. Effective SRL interventions should account for the broader classroom context, including student engagement in rich, demanding tasks (Dignath & Veenman, 2021; Perry, 1998; Perry & VandeKamp, 2000). As Paris and Paris (2001) noted, whether students use self-regulation tactics in school, what kinds of strategies they use, how they are rewarded for their use, and how much effort they expend being regulated and strategic, depends on the tasks and contexts that teachers create for students (p. 93).

Meta-analyses have shown that the most successful outcomes arise when strategy instruction is paired with motivational and contextual supports (Dignath et al., 2008; Dignath & Büttner, 2008; Dignath & Veenman, 2021; Hattie et al., 1996), which increase the likelihood that students will apply the taught strategies (see also Efklides, 2011; McCombs & Marzano, 1990).

The integration of strategy promotion with content learning is a serious challenge for SRL professional development studies. Studies of teachers’ educational beliefs indicate that teachers often consider the teaching of subject content as different and unrelated to the teaching of learning strategies (Lawson et al., 2023; Vosniadou et al., 2021). A very common justification for not spending enough time on SRL promotion is that teaching content is more important than teaching SRL strategies and therefore should come first (Vosniadou et al., 2021). Such responses indicate the difficulties inherent in the application of a 21st century education curriculum that combines student cognitive activation and conceptual understanding with the development of student SRL competencies.

In an interesting study, Pauli et al. (2007) investigated whether math teachers were successful in combining “more open forms of instruction, which are forms primarily at the surface level of lesson organization” with “quality-oriented instruction that aims at fostering conceptual understanding and problem-solving processes” (p. 295), in agreement with the goals of the 1990’s educational reform in Switzerland. They used video analysis of math lessons from the TIMMS study (Hiebert et al., 2005), surveys of teachers and students, and assessments of student math understanding with a sample of 79 teachers and 1407 8th grade students. Analysis using structural equation modeling revealed no meaningful link between SRL and students’ ability to solve problems independently. While teachers’ constructivist beliefs strongly influenced their support for independent problem solving, they did not significantly impact SRL promotion. The researchers emphasized the need for targeted educational strategies that equally prioritize both SRL and conceptual understanding; without this balance, instructional reforms risk remaining surface-level and failing to transform deeper teaching practices.

A difficulty in designing interventions that address both the direct and indirect promotion of SRL is that of defining and measuring the characteristics of a classroom learning environment that supports SRL indirectly. Perry (1998) and Perry and VandeKamp (2000) linked successful SRL development in children to their involvement in rich, purposeful, and demanding learning activities. Dignath and Veenman (2021) argued that SRL is best fostered in constructivist classrooms, where educators encourage students to connect new ideas with prior knowledge, tackle open-ended problems with multiple solutions, and engage with content presented in a relevant and meaningful way.

In previous work, we argued that the Interactive, Constructive, Active, Passive (ICAP) theory of cognitive engagement from Chi and Wylie (2014) offers a valuable lens for understanding how SRL can be indirectly supported within classroom environments (Vosniadou et al., 2024) because it provides clear and measurable criteria for defining the different modes of student cognitive engagement and links them to distinct learning processes and outcomes. According to ICAP, students can engage with tasks in passive, active, constructive, or interactive modes. A passive mode of engagement is one where learners are not engaged in any observable behaviors related to learning (like listening or watching), whereas an active mode of engagement requires some observable behaviors, like reading, underlining certain text sentences, or identifying main ideas. Constructive engagement requires behavior that generates new ideas that go beyond the information given, such as comparing, identifying similarities and differences, or explaining the meaning of a sentence in their own words. During this process, learners go beyond the information provided by generating inferences that are not explicitly stated in the text. Interactive engagement requires interaction between two or more agents where both partners make substantive contributions at the constructive mode to the topic under discussion that result in the improvement of their understanding beyond the level attained by the individual members of the group (Chi, 2021). The ICAP theory argues that constructive and interactive modes of engagement are associated with better learning outcomes than passive and active ones.

The ICAP theory has useful implications for SRL teacher professional development and could have beneficial effects for the way that SRL interventions are promoted and delivered in class situations (see Chi & Boucher, 2023; Chi et al., 2023). For example, according to ICAP, a given learning strategy, such as note-taking, can result in better learning outcomes if it is used in the context of constructive and interactive tasks compared to active tasks.

We suggest that the use of ICAP theory as an index of indirect strategy promotion, in combination with observational measures of direct strategy promotion, would enable consideration of both forms of promotion in a PDP. This approach has been adopted in the current research.

### 1.2. The Present Study

The PDP lasted for 7 months and consisted of two parts. In the first part, the teachers were presented with information about the ICAP theory. During this part, the teachers were encouraged to include more constructive and interactive tasks in their classroom and fewer passive and active ones. The second part of the intervention presented the teachers with information about SRL emphazing the provision of knowledge about SRL, the use of explicit strategies, and explanations about the benefit of strategy use.

In both parts of the PDP, the participating teachers were encouraged to actively participate in discussions with the researchers and with the other teachers during face-to-face or online sessions and to express their views. The content of the PDP included excerpts from videos of classroom instruction which the participants viewed to identify instances of passive, active, constructive, and interactive tasks and/or examples of explicit and implicit strategy promotion, promotion of SRL knowledge and beliefs, and metacognitive support and reflection. In the case of the ICAP part of the PDP, the teachers were provided with a simplified ICAP coding guide (see Table 1) and were instructed on how to use it to analyze the transcript from a videoed lesson. Finally, each teacher was provided with a transcript of the video of her/his own classroom teaching taken before the start of the PDP and was encouraged to examine and reflect on it.

Fourteen teachers participated in the first part of the PDP, but only nine teachers continued with the second part, which took place during COVID-19-related disruptions in schools. The investigators observed and videotaped the participants’ classroom teaching before the PDP (Round 1), after the ICAP part of the PDP (Round 2), and again after the SRL part of the PDP (Round 3).

All teacher and student speech was transcribed verbatim, and information was also included about teacher and student actions deemed necessary to understand the context in which the discourse took place. The effects of the PDP on the teachers’ practices were investigated through an analysis of the talk and actions of the teachers in the transcripts of the videos taken from each teacher’s classroom during Rounds 1, 2, and 3.

To evaluate the influence the PDP had on the indirect promotion of SRL, the transcripts were analyzed using an ICAP-based coding guide (ICAP-CG) developed in previous work (Vosniadou et al., 2023). The ICAP-CG was applied to analyze the types of lesson activities teachers assigned and assess the level of cognitive engagement they encouraged by reviewing how teachers instructed students to carry out those tasks (see Table 1).

The influence of the PDP on the direct promotion of SRL was examined through a separate analysis using an observation protocol based on the SRL teacher promotion framework (TPF) (Vosniadou et al., 2024). The SRL TPF observation tool was employed to evaluate teachers’ spoken contributions, identifying whether they involved direct or indirect strategy instruction, supported SRL-related understanding and beliefs, encouraged reflective thinking, or offered metacognitive support. When an utterance was classified as promoting SRL, further analyses determined its specificity to the subject matter, the SRL skill addressed, the method of delivery, and whether the teacher highlighted the strategy’s advantages (see Table 2, p. 11).

We hypothesized that the first part of the intervention would be deemed successful if we obtained increases in the proportion of constructive and interactive tasks or the overall time spent on such tasks, and decreases in the proportion of or overall time spent on passive and active tasks in the participants’ lessons from Round 1 to Round 2. Although passive and active tasks are of course relevant during a lesson, we expected that after the PDP the teachers would understand the value of constructive and interactive lesson tasks, thus increasing the time spent on them. No specific benchmarks were set. We expected these changes to be observed in Round 3, which provided a delayed test for the success of the ICAP part of the intervention.

Regarding the second part of the investigation, we predicted an increase in the proportion of instances of explicit strategy promotion, explanations of the benefit of use of the strategies, and knowledge about SRL in general. The research questions were the following:

(1) (A) Will we observe a decrease in the proportion of and/or overall time spent on passive and active tasks and increase the proportion of and/or overall time spent on constructive and interactive tasks in the participants’ lessons from Round 1 to Round 2? (B) If so, will the changes also be observed during Round 3? (C) Will the changes be observed for all teachers?
(2) (A) Will we observe an increase in the proportion of explicit strategy promotion, knowledge about SRL and explanation of the benefits of SRL in teachers’ talk to students during lessons from Round 1 and Round 2 to Round 3? (B) If so, will these changes be observed in the case of all teachers?

## 2. Materials and Methods

### 2.1. Participants

The participants were 14 teachers from five schools, all in the Greater Adelaide region of South Australia. All but one of the teachers taught in secondary schools (Year 7 to 12), the exception being a combined Year 1 and 2 class teacher. This teacher was included because she was particularly eager to participate in the PDP and was also interested in teaching science subjects. In addition, we wanted to include as many teachers as possible in the study.

All except one teacher taught in public schools. The teachers were between 24 and 57 years of age (*M* = 39), with 3 of them having more than 25 years of teaching experience. Most participants were teaching classes broadly classified as STEM subjects. More specifically, five teachers taught Mathematics lessons, six teachers taught hard sciences (including Physics, Biology, Science, and Aviation), and two teachers taught soft science lessons (Food Technology). Approval for the research was granted by the Human Research Ethics Committees of Flinders University. Participants provided informed consent to participate in all aspects of the PDP.

### 2.2. Materials

Prior to commencing the PDP, teachers completed a pre-test including demographic items. The researchers videotaped one classroom observation for each teacher prior to the start of the PDP, a second at the end of Round 1, and a third at the end of Round 2 of the intervention. The focus of this paper is on the analysis of the discourse and relevant action of the teachers and the students as recorded in the transcripts of the video observations.

The PDP materials consisted of two ICAP modules and two SRL modules: ICAP Module 1 described the ICAP theoretical framework and the modes of student cognitive engagement and provided examples of activities that belonged in the different modes of student cognitive engagement with an emphasis on the constructive and interactive modes. ICAP Module 2 provided information and guidelines about the application of ICAP in instruction and the ICAP-CG. It included a coding exemplar so that the participants could understand how to apply the ICAP-CG to score lesson tasks and then prompted participants to use it to evaluate the transcript of an excerpt from a videoed lesson. The teachers were also provided with a transcript of the video observation of their own lesson and were encouraged to score it and reflect on it.

SRL Module 1 explained key aspects of SRL theory and strategies and outlined their benefits. The participants were asked to provide examples of instruction promoting students’ ability to better plan their learning. Module 2 described the different SRL capabilities and the different types of SRL strategy promotion and provided examples and excerpts from videoed observations demonstrating the promotion of explicit strategies, implicit strategies, knowledge and beliefs about learning, metacognitive support, and metacognitive reflection based on the SRL/TPF observation protocol. At the end of the module, the participants were asked to provide an example of a lesson aimed at introducing students to a new concept relevant to their current curriculum, describe details of SRL promotion, and justify the methods of promoting it.

### 2.3. Procedure

Researchers contacted principals of nearby schools with a letter outlining the study’s objectives and requesting approval to recruit teachers. Upon receiving consent, they distributed information about the study to all teachers at the school, inviting volunteers. Interested teachers signed a consent form, completed a demographic questionnaire, and coordinated with the research team to join the PSP sessions. All participants were guaranteed confidentiality and reminded that their involvement was entirely voluntary.

Videos of the participants’ classrooms were taken three times during the PDP: before the beginning (Round 1), after the completion of the indirect SRL/ICAP part of the PDP (Round 2), and after the completion of the direct SRL part of the PDP (Round 3). Seven of the participating teachers did their own filming; the remaining were filmed by one of the researchers. The teachers who did their own filming were provided with all the required equipment and were given detailed instructions on how to make the videos. To capture audio clearly, teachers wore lapel microphones throughout the recordings, allowing researchers to hear their speech during both full-class teaching and while interacting with students during independent work.

Five sessions with the participating teachers occurred during the intervention. Session one occurred in person following completion of the pre-test. It was used to welcome the participants, explain the purpose of the study, and introduce them to the indirect SRL/ICAP part of the intervention. Session two occurred online two months later following the filming of the participants’ first lesson and subsequent completion of the SRL/ICAP online modules. During this session, the researchers answered the participants’ questions related to these modules and the application of the ICAP coding guide to analyze transcripts of videoed observations. The participants were encouraged to use the ICAP coding guide to analyze the transcript from the video observation of their own classroom and reflect on it. Session three occurred in person two months later following the filming of the participants’ second lesson. During this session, the participants were introduced to the direct SRL promotion part of the intervention. Session four occurred online one month later following the filming of the participants’ third lesson. The final session to acknowledge participants’ contributions occurred in person several weeks later. The in-person sessions lasted three hours each. The online sessions lasted as long as needed, usually two hours. All sessions took place in a university meeting room. All teachers participated in all sessions and completed all mandatory tasks.

Five of the 14 teachers did not participate in Round 3 of the study; this reduced the number of participants to 9. The main reason for this attrition was the increased workload and stress levels of the teachers due to COVID-19 restrictions, which at that time started to be in effect in schools in South Australia. While most classrooms still had face-to-face lessons, there were some occasional shutdowns and the teachers were preparing for online classes.

### 2.4. Video Transcription and Scoring

The videoed observations taken from the participants’ classroom lessons were transcribed and analyzed for changes in the ICAP modes of cognitive engagement and forms of SRL promotion in the teachers’ speech and action overtime. Five of the videos were first transcribed by two of the researchers independently. All teacher and student speech was transcribed verbatim, and information was also included about teacher and student actions deemed necessary to understand the context in which discourse took place. After agreeing to standard procedure for the transcriptions, and after achieving a good level of coding reliability using Cohen’s Kappa scale, the remaining videos were transcribed by one researcher.

Due to variations in lesson duration, researchers chose to analyze the initial 40 min of each recorded session. Two lessons were shorter—one lasted 38 min and another only 20 min. Transcripts from each session were inserted into an Excel coding template, which included minute-by-minute timestamps to track the length of each instructional event.

The transcripts were scored for the indirect promotion of SRL using the ICAP-CG and for the direct promotion of SRL using the TPF-SRL observation protocol. Both scoring guides were developed and are described in detail in previous work (Vosniadou et al., 2023, 2024).

*Indirect SRL Promotion*—ICAP-CG. The coders viewed the filmed lesson, read the lesson transcript, determined the lesson tasks, and proceeded to determine the mode of student engagement the teachers promoted using the ICAP-CG described in Table 1. An additional mode of cognitive engagement—active/collaborative—was added because the teachers sometimes gave instructions to students to work in a collaborative, interactive way but did not use constructive verbs to describe the task. In these cases, the mode of cognitive engagement was described as active/collaborative.

Lesson tasks were segmented into one-minute intervals. During each minute, researchers highlighted all verbs used by the teacher to present or explain the task to the class. These verbs helped to identify the dominant mode of student cognitive engagement for that time frame. To determine the overall engagement type for each task, codes from each minute were tallied, and the most frequently occurring code was assigned to the entire task.

*Direct SRL Promotion*. All teacher utterances were examined against the criteria of the SRL/TPF for SRL promotion type described in Table 2. Every utterance determined to represent a promotion type, namely explicit instruction, implicit instruction, promotion of knowledge/beliefs, metacognitive reflection, or metacognitive support, was marked as such. The utterances that represented a promotion type were examined again to determine the capabilities being promoted, the manner of promotion, the presence of benefit of use information, and whether it was domain-general or domain-specific.

*Reliability*. Initially, four researchers analyzed six lesson transcripts, applying SRL and ICAP codes where relevant. They reviewed discrepancies and adjusted the coding guidelines as needed. Once consensus was reached, two researchers independently coded ten additional transcripts. The inter-rater reliability assessment showed an 84% agreement rate, with a Cohen’s Kappa of 0.68 for direct SRL promotion and 93% agreement with a Cohen’s Kappa of 0.86 for indirect SRL promotion (ICAP). The remaining transcripts were coded by a single researcher.

## 3. Results

*Intervention Part 1*—*Indirect Promotion of SRL-SRL/ICAP*. The transcripts of the participants’ speech and action during the lessons in all three rounds were examined to determine the overall number of tasks the teachers designed. As can be seen in Table 3, there was a decrease in the median number of lesson tasks the teachers designed from Round 1 (5) to Rounds 2 and 3 (3).

The unit of analysis in this part of the investigation was the lesson task. Each lesson task was examined using the ICAP-CG to answer the research questions related to the indirect promotion of SRL. Table 4 shows the frequency and percent of the task engagement modes assigned to the participating teachers during the first part of the intervention, and Table 5 shows the frequency and percent of minutes of instruction time the participating teachers spent in each engagement mode. As expected, the frequency of passive and active tasks in these modes of cognitive engagement decreased from Round 1 to Round 2 (Research question 1a). An unexpected finding was that the number of constructive tasks also decreased, while there was an increase in the frequency of interactive tasks from Round 1 to Round 2. The teachers clearly preferred designing interactive compared to constructive tasks; however, given that the definition of interactive in the ICAP context also includes constructive, the overall number of constructive student cognitive engagement increased from 18 tasks in Round 1 (24%) to 20 in Round 2 (46%).

Because the overall number of lesson tasks decreased from Round 1 to Round 2, the minutes that the students spent in the combined constructive and interactive engagement modes is a better measure of the observed changes than the frequency of the different modes of engagement. As can be seen in Table 5, the time of student cognitive engagement in constructive and interactive modes increased from 130 min in Round 1 to 249 min in Round 2, while the time of engagement in passive and active modes decreased from 406 in Round 1 to 278 min in Round 2.

Wilcoxon signed-rank tests were conducted to compare the proportion of lesson task modes of engagement (passive, active, and active collaborative, constructive, and interactive). For each mode of engagement, the test examined within-participant changes across the three rounds (Table 6). Median differences were calculated to determine the direction and magnitude of change. Holm-adjusted *p*-values were used to account for multiple comparisons. The results indicated that the proportion of tasks in the combined passive, active, and active/collaborative engagement modes decreased from Round 1 to Round 2 while the proportion of the combined constructive and interactive tasks increased from Round 1 to Round 2 (Research question 1a). The increase in the number of combined constructive and interactive tasks was also observed when Round 1 was compared to Round 3. The proportion of constructive and interactive modes of engagement in teachers’ tasks did not change between Rounds 2 and 3, confirming the maintenance of the effect of the intervention (Research question 1b). Although the changes were in the expected direction for all these comparisons, only the increase in constructive and interactive tasks from Round 1 to Round 2 was statistically significant (V = 0, *p* (Holm) < 0.001, r = 0.88, 95% CI [0.88, 0.88]) with median proportion difference of 0.22 (see Table 6). This change suggested a very strong, consistent increase in constructive and interactive tasks from Round 1 to Round 2, indicating a substantial change in teaching behavior.

**Figure 1:**
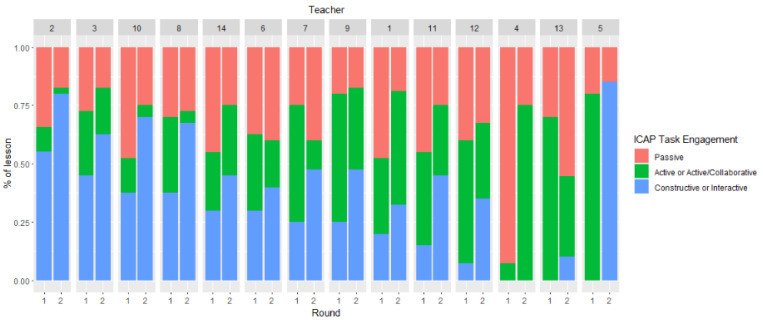
Proportion of the tasks in the passive, combined active and active/collaborative, and combined constructive and interactive engagement modes of the 14 teachers in Rounds 1 and 2. *Note*: A total of 13/14 teachers increased the number of constructive/interactive tasks from R1 to R2.

**Figure 2:**
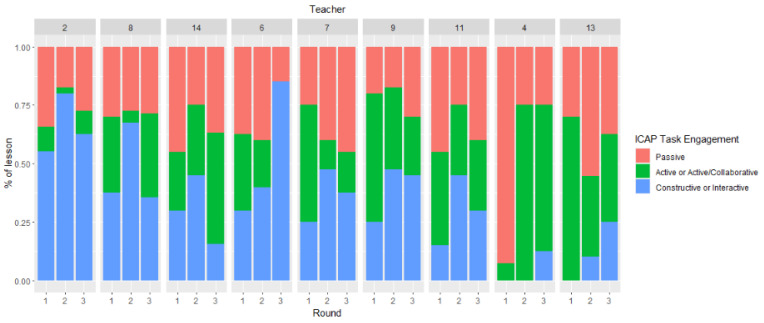
Proportion of tasks in the passive, combined active and active/collaborative, and combined constructive and interactive engagement modes of the 9 teachers who participated in Rounds 1, 2, and 3. *Note*: A total of 7/9 teachers increased the number of constructive/interactive tasks from Round 1 to Round 3.

To answer Research question 1c regarding possible teacher differences, we examined the proportion of tasks in the passive, combined active and active/collaborative, and combined constructive and interactive engagement modes for the 14 participating teachers in Rounds 1 and 2, and for the 9 participating teachers in Rounds 1, 2, and 3. As shown in Figure 1 and Figure 2, the results were in the expected direction for all teachers. Thirteen teachers increased the number of combined interactive and constructive tasks in their lessons from Round 1 to Round 2, while one teacher did not design any interactive or constructive tasks but rather increased the number of active and active/collaborative tasks. As noted earlier, a few of the tasks the teachers designed were interactive and active—not constructive—and therefore were coded as active/collaborative. An increase in the number of interactive and constructive tasks was also observed in Round 3 compared to Round 1 for most of the nine teachers who participated in the study’s third round. Figure 1 and Figure 2 show the proportion of tasks, taking into consideration the length of the observation, in the different modes of cognitive engagement.

*Intervention Part 2*—*Direct Promotion of SRL*. The transcripts were examined again using the SRL/TPF observation protocol to obtain information regarding the direct promotion of SRL. The unit of analysis in this part was the teachers’ utterances. Each utterance was examined to determine if it represented a case of SRL promotion and if so what type of promotion was involved—namely, explicit strategy, implicit strategy, promotion of SRL knowledge or beliefs, metacognitive support, or metacognitive reflection. If an utterance was placed in one of the above-mentioned types of SRL promotion, it was further examined to determine which capabilities it promoted, whether the promotion was domain-specific or domain-general, and whether it included information about the benefit of use of the strategy.

The average number of utterances per teacher found to represent an instance of SRL promotion was 49.3 for Round 1, 56.9 for Round 2, and 51.8 for Round 3. Table 7 shows the frequency and percentage of SRL promotion related to each of these SRL utterances for Rounds 1, 2, and 3. The direct SRL promotion intervention took place after Round 2, so we expected increases in the percentage of explicit strategy promotion, promotion of SRL knowledge and beliefs, and benefit of use from Round 2 to Round 3 (Research question 2a).

Wilcoxon signed-rank tests with Holm-adjusted *p*-values were conducted to compare the proportion of different SRL promotion categories between rounds (see Table 8). Although the frequencies of types of promotion were all in the expected direction, there were statistically significant increases only in utterances promoting motivational capabilities (V = 11, *p* (Holm) < 0.05, r = 0.7, 95% CI [0.33, 0.88], median proportion difference of 0.05) and utterances indicating that the SRL promotion was domain-general rather than domain-specific (V = 3, *p* (Holm) < 0.05, r = 0.83, 95% CI [0.63, 0.88], median proportion difference of 0.08) strategies between Rounds 1 and 2. Both results suggest a very strong, consistent increase in tasks promoting motivational capabilities and domain general SRL promotion from Round 1 to Round 2, indicating a substantial change in teaching behavior.

**Figure 3:**
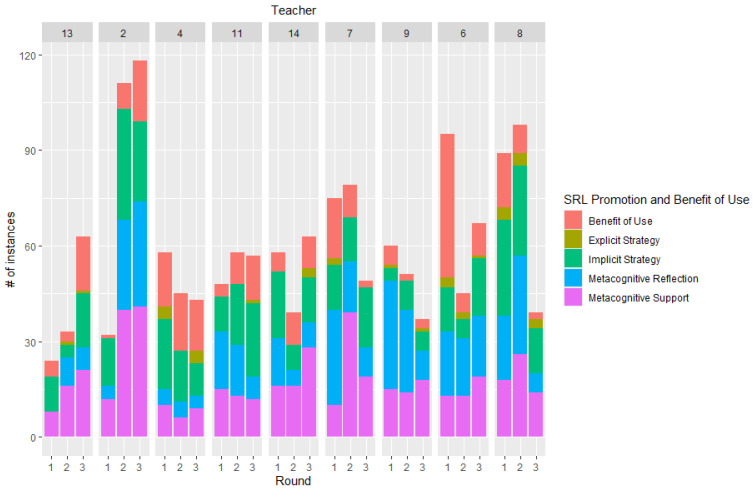
Proportion of SRL promotion and benefit of use utterances for the 9 participating teachers in Rounds 1, 2, and 3.

To answer Research question 2b, regarding teacher differences, we examined the results for the nine teachers who participated in Round 3, shown in Figure 3. These figures show different types of changes in instruction amongst these teachers. Teachers 13, 2, 11, and 14 showed increases in the number of utterances representing the benefit of use of the strategies being promoted. In the case of teachers 11 and 14, we observed a slight increase in the number of explicit strategies, whilst teacher 6 increased the number of implicit strategies.

As mentioned earlier, five teachers did not participate in Round 3 or the PDP. To assess whether this attrition may have biased results, a sensitivity analysis compared baseline (Round 1) proportions of coded behaviors between teachers who were retained (n = 9) and those who discontinued participation after Round 2 (n = 5). Wilcoxon rank-sum tests were conducted across 23 ICAP and SRL categories.

Of the 23 categories, 22 showed no evidence of baseline differences between retained and dropped participants (all *p* values > 0.10). A single significant difference was observed for SRL Capabilities—Resource Management (*p* < 0.05), indicating a somewhat higher initial frequency of this behavior among teachers who continued to Round 3. Given the small sample size and the number of comparisons performed, this isolated result is likely due to chance variation rather than systematic attrition bias. Overall, the pattern suggests that teacher drop-out had minimal impact on the representativeness of the Round 3 sample or on the robustness of the direct-promotion findings.

## 4. Discussion

A PDP was designed to provide secondary school teachers with information relevant to both indirect and direct promotion of SRL in the classroom and investigate its effects on the teachers’ classroom practices. The measures used as indices of the indirect promotion of SRL were based on the ICAP theory (Chi & Wylie, 2014) and emphasized the importance of interactive and constructive lesson tasks as a means of increasing student cognitive engagement. The direct SRL promotion measures were based on the SRL TPF (Vosniadou et al., 2024) and highlighted the importance of explicit promotion of SRL knowledge and strategies and the benefits of these strategies. Other noticeable features of the PDP were the introduction to teachers of theories and frameworks related to SRL and to procedures for both indirect and direct promotion of SRL strategies. An important aspect of the PDP was the introduction of scoring guides for ICAP and their use in evaluating and reflecting on teacher practices, including their own, from the transcripts of the video observations. In addition, the PDP encouraged the active participation of the teachers in face-to-face and online discussions with the researchers and amongst themselves and the provision of examples and video excerpts of real classroom lessons. Three video observations of the participants’ classrooms were made, before the PDP (Round 1), after the SRL/ICAP indirect promotion part (Round 2), and after the direct SRL promotion part (Round 3).

*Indirect SRL Promotion: The ICAP-based part of the PDP.* The examination of the teachers’ classroom practices indicated a clear effect of the ICAP-based part of the PDP that provided a positive answer to the first research question. Namely, the teachers designed fewer passive and active lesson tasks and increased the number of, and time spent on, interactive and constructive learning tasks at Round 2 compared to Round 1. This increase in the time spent on interactive and constructive tasks maximized the students’ opportunities for use of knowledge change processes that is likely to increase the generative power of their learning actions (Fiorella, 2023; Wittrock, 1989). Through the changes they made to the design of their lesson tasks in Round 2, the teachers provided their students with classroom environments that would be more likely to stimulate more effective knowledge construction. The teachers continued to produce more interactive and constructive lesson tasks in their classrooms even after the second SRL promotion part of the PDP (Round 3) compared to their pre-intervention practices (Round 1), indicating that the ICAP intervention had a lasting effect. The increase in the number of, and time spent on, interactive and constructive lesson tasks was observed in the case of all teachers. In interviews conducted with some of the teachers as part of a larger research project, a teacher reported on the interactive tasks that he introduced his students to.

But they seem to have a lot more fun doing it because they’re working with each other. They can share their ideas, and it wasn’t just alright. I’ve got to sit here on my own and come up with a bunch of ways I can test, and I’ve got to run the tests. It was Let’s all come up with these ideas together and then we can help each other do the testing and take photos of each other’s work while we’re running tests, stuff like that.

The results of the first part of the intervention showed that the teachers preferred to design tasks that were interactive rather than only constructive. This finding suggests that interactive tasks might be perceived by the teachers as best representing active cognitive engagement on the part of the students. Given that interactive tasks are also constructive, these two types of lesson tasks were very similar, with the only difference being whether they were performed individually or in interaction with other students. They usually involved solving problems individually or in groups, finding information for a research paper, designing an experiment, or discussing its results, critically evaluating a text, self-explaining or explaining ideas to another student, generating ideas in whole-class discussion, or debating ideas in a group. Overall, the results resembled those observed in other intervention studies with ICAP (Chi et al., 2018; Stump et al., 2018).

While passive and active tasks have a place in the classroom, constructive and interactive tasks have greater possibilities for engaging students in activities that require generative processing of information for increased understanding. Such tasks also offer students more opportunities to exercise their cognitive, metacognitive, affective, motivational, and resource management capabilities. In contrast, active tasks such as filling out forms or work sheets, completing calculations, taking verbatim notes while reading or watching a video, or engaging in class discussions involving recalling information are less challenging both cognitively and in terms of being likely to stimulate more effective SRL competencies. Research has provided ample evidence for the positive effects of constructive and interactive learning tasks on students’ learning and achievement (Chi & Wylie, 2014; Coleman et al., 1997; Gobert & Clement, 1999; Menekse et al., 2013).

An unexpected effect of the ICAP intervention was its influence on the teachers’ SRL. There was a statistically significant increase in the number of teacher statements that were domain-general compared to domain-specific and statements that promoted motivational capabilities in Round 2 compared to Round 1. In other words, the change in the learning environment observed after the introduction of ICAP had an impact on the teachers’ SRL promotion before the teachers were exposed to information about SRL. This influence was not in the direction of including more explicit SRL strategies. Rather, as the teachers spontaneously increased their motivational statements. Most of these motivational statements were associated with metacognitive support. In other words, the teachers supported the students for their effort and encouraged them to try to maintain their current learning actions (e.g., “you are doing good work” or “try again”). A practical consequence of this action by teachers is likely to be students’ maintenance of their use of more generative learning actions, though we do not have information that would indicate that such teacher action was deliberately directed at such generative action on the part of the students.

These results agree with previous ICAP studies showing that teachers who designed constructive and interactive lesson tasks were likely to give students more autonomy over their learning, but did not deliberately engaging in direct SRL promotion through the explicit or implicit teaching of SRL strategies or the promotion of knowledge about SRL (Vosniadou et al., 2024; see also Pauli et al., 2007). However, it must be recognized that on its own the provision of increased levels of autonomy will not stimulate increased levels of cognitive engagement. Other observational studies have also shown that teachers who may be good at giving students freedom of choice about their learning do not usually teach them how to manage their autonomy (Bolhuis & Voeten, 2004). 

Regarding the direct promotion of SRL, a question of interest is whether this situation changed after the second part of the intervention which introduced information about SRL and the importance of its direct promotion through the explicit teaching of SRL knowledge and strategies. Our hypothesis was that we would observe an increase in the instances of explicit promotion of SRL knowledge and strategies and explanations of their benefit of use. Indeed, the analysis of Round 3 of the videoed observations of the teachers’ classrooms showed an increase in explicit and implicit SRL strategies and knowledge/belief statements in Round 3 compared to Rounds 1 and 2. These effects were not the same for all participants. Some teachers increased their teaching of explicit and implicit strategies while some focused more on SRL knowledge and its benefits. Although these results were not statistically significant, they indicate that the direct SRL promotion part of the PDP had some positive impact on the participating teachers’ instruction.

The impact of the direct SRL promotion part of the PDP on the teachers’ practices was not as pronounced as that for the ICAP intervention. One possible reason was the reduced number of teachers during Round 3, and, more generally, the effects of COVID-19, which were starting to influence classrooms in South Australia at the time of the SRL intervention. Another possible factor was the decrease in the amount of time spent on the direct SRL promotion part of the program compared to the time spent on the indirect promotion. Because less time was spent on this part of the program, the teachers did not use a simplified SRL/TPF coding guide to evaluate transcripts of videoed classroom observations, including observations of their own classroom, regarding the direct promotion of SRL, as was the case with the ICAP part of the PDP.

It could also be argued that the ICAP part of the PDP was easier for teachers to implement than the SRL part of the PDP. With ICAP, the teachers had to learn to distinguish between passive, active, constructive, and interactive lesson tasks and to increase the number and time spent on interactive and constructive ones. The SRL PDP, on the other hand, was more complex. It introduced distinctions between the promotion of knowledge vs. strategies, of explicit vs. implicit strategies, of the inclusion of benefit of use, and of cognitive, metacognitive, motivational, and affective strategies, amongst others.

The above suggest that future programs could profit from simplifying the direct promotion of SRL, maybe by focusing more on the explicit promotion of SRL knowledge and strategies with the inclusion of information about their benefit of use. More examples indicating use of explicit strategies in the application of constructive and interactive lesson tasks would also be helpful. The idea of using ICAP and SRL coding guides to evaluate transcripts of videoed classroom observations, which the current PDP introduced, also proved to be very useful and particularly the part in which the teachers evaluate and reflect on their own lessons. This would mean simplifying the SRL coding guide, including more examples of its use in the context of constructive and interactive tasks, and giving teachers more time to use it to evaluate real instruction, including reflecting on their own.

Nevertheless, some of the teachers found interesting ways to combine interactive and constructive tasks with the promotion of explicit strategies. One of the teachers introduced an interactive task but also explicitly taught his students strategies of how to interact effectively. Another teacher introduced a self-explanation strategy in his Year 8 Algebra class using an interactive task in which the students had to state explicitly the strategy they used and explain its benefits. The following are some of the comments made by one of the teachers reflecting on the program.

Students were not just remembering how to do it because it was rote learned. They were remembering the area model of multiplication and how that connected to it. And then we asked students to actually explain that in their test. And we found that their communication overall improved a lot through that explicit teaching of how to communicate and explain their steps.

### Limitations and Future Directions

One limitation of the research was the small number of participants, especially those in the second part of the PDP. The study needs to be replicated with more lessons videotaped at each round. Recording multiple lessons would have ensured greater accuracy in our assessment. The study also did not address the role of the students or the interaction between students and teachers, which is a topic for future research. More studies are also needed outside the Australian context and STEM classes and encompassing more grades to test the transferability of the findings.

Another limitation was that less time was spent on the direct SRL promotion part of the study than the first part. It is possible that a PDP with a larger number of participants, more time spent on direct SRL promotion, with a simplified SRL coding guide, in non-COVID-19 times would have produced better results. Although some of the teachers were very creative in the way they combined the design of interactive and constructive tasks with the explicit teaching of SRL strategies and the promotion of knowledge about SRL, the PDP could be improved with the inclusion of more explicit examples for the teachers to consider, opportunities for teachers to use the SRL scoring guide to evaluate and reflect on their own teaching, as well as a more explicit discussion of these issues during the face-to-face and online sessions.

## 5. Conclusions

A PDP was designed to combine the indirect and direct promotion of SRL with its indirect promotion based on the ICAP theory and to measure its impact on teachers’ actual practices. The results showed statistically significant effects on teachers’ indirect promotion of SRL specifically on their inclusion of more interactive and constructive tasks in their lessons and fewer passive and active tasks. This effect can be seen to have provided students with increased opportunity to activate more generative learning actions that could help them to construct more complex understandings. The teachers’ direct SRL promotion also increased, first in the direction of more motivational, metacognitive support statements encouraging students to keep on trying, and secondly, by paying more attention to the explicit teaching of SRL strategies and the benefits of their use. Overall, the results showed that it is possible to design interventions that aim at combining teaching for understanding with the direct promotion of SRL strategies.

## Figures and Tables

**Table 1:** The ICAP coding guide.

Mode of Cognitive Engagement	Overview	Corresponding Verbs (Noted in Teacher’s Whole-Class Instructions or Observed in Students’ Behavior)
Passive	Learners are oriented toward and receive information from instructional materials without overtly doing anything else related to learning.	Engage, Go through, Listen, Look, Observe, Read, etc.
Active	Requires some form of motoric behaviors that cause focused attention.	Add, Annotate, Break down, Calculate, Categorize, Check, Choose, Circle, Click, Complete, Copy, Cover, Cross out, Delete, Describe, Expand, Fill in/out, Find, Fold, Guess, Identify, Include, Keep/take notes, Label, List, Match, Measure, Move, Name, Number, Order, Paraphrase, Pick, Place, Plot, Practice, Re-organize, Recall, Record, Refer to, Review, Round to, Show, Type, Use, etc.
Active/Collaborative	Requires collaboration between two or more partners that does not meet the criteria established further below for the interactive mode of cognitive engagement.	Active verbs as listed above
Constructive	Requires students to produce additional outputs or products beyond those provided in the learning materials. It requires actions and generates new ideas that go beyond the information given.	Ask questions, Brainstorm, Build, Come up, Comment, Compare, Connect, Construct, Create, Decide, Defend, Determine, Draw, Explain, Generate, Graph, Justify, Predict, Put/explain/write in own words, Represent, Set goal, Sketch, Solve, State, Suggest, Summarize, Support, etc.
Interactive	Requires collaboration among two or more partners that meets two criteria: Both partners’ utterances must be primarily *constructive*; A sufficient degree of turn taking must occur. Generates knowledge beyond the original learning materials and beyond what the partner has said; both partners need to be *constructive.*	Constructive verbs as listed above, but in *pairs or small groups*. Interactive verbs that elicit co-constructive engagement: Argue, Ask/Answer each other’s questions, Build upon, Correct, Critique, Debate, Defend, Elaborate, Explain, Justify

**Table 2:** Direct SRL promotion coding guide.

SRL Promotion Types	SRL Capabilities	Domain	Maner of Promotion	Benefit of Use
**Explicit strategy:** A strategy is explicitly taught using the word strategy or naming the strategy **Implicit strategy:** A strategy or procedure is taught without using the word strategy or naming it **Knowledge/beliefs:** Information is provided about the nature and/or processes of learning, and/or beliefs about learning and their impact on academic performance **Metacognitive reflection:** Students are asked to reflect on, monitor, or evaluate their learning **Metacognitive support:** Students are prompted to remember and use relevant information or learning strategies	**Cognitive:** Promotion related to mental actions and brain processes related to learning (attention, task analysis, rehearsal/retrieval, imagery, knowledge storage, elaboration, organization, problem solving). **Metacognitive**: Promotion related to learners’ knowledge and control of their cognition (planning, prediction, monitoring, evaluation) **Motivational:** Promotion related to what stimulates a person to act to achieve a desired goal (goal setting, interest, self-efficacy, attributions, rewards) **Affective**: Promotion related to the feelings/emotions emerging from one’s actions and their impact on learning (anxiety, excitement, anger, embarrassment) **Resource management:** Promotion related to ways of organizing the physical (space, resources) or social environment (collaboration, seeking help from others, avoiding distractions)	**General:** Promotion related to several subject areas **Specific**: Promotion related to specific subject areas	**Direct verbal:** Teacher tells students something directly **Modelling:** Teacher shows students how to do something (often with verbal instructions as well) **Prompting:** Teacher provides a hint, cue, or encouragement, or asks a question	**Explanation:** Teacher explains the benefit of the strategy or knowledge to support student understanding

**Table 3:** Number of lesson tasks used by the participating teachers.

Round	Tasks		
	Min	Median	Max
1	2	5	8
2	1	3	7
3	1	3	6

**Table 4:** Frequency and percentage of the modes of engagement assigned to the participating teachers’ lesson tasks (n = 14).

Mode of Engagement	Round 1	Round 2	Round 3
Passive	28 (39.4%)	11 (22%)	10 (33.3%)
Active	25 (35.2%)	17 (34%)	9 (30%)
Active/Constructive	0	2 (4%)	0
Constructive	10 (14.1%)	5 (10%)	1 (3.3%)
Interactive	8 (11.3%)	15 (30%)	10 (33.3%)
Total	76	50	30

**Table 5:** Frequency and percentage of time (minutes) of the modes of engagement assigned to the participating teachers’ lesson tasks.

Mode of Engagement	Round 1	Round 2	Round 3
Passive	210 (39%)	146 (26.7%)	110 (31.8%)
Active	198 (36.4%)	151 (24.2%)	101 (26.6%)
Active/Collaborative	2 (0.4%)	19 (3.5%)	9 (2.6%)
Constructive	69 (12.8%)	55 (10.1%)	21 (6.1%)
Interactive	61 (11.3%)	194 (35.5%)	114 (32.9%)
Total	538	546	346

**Table 6:** Wilcoxon signed-rank tests (Holm-adjusted) for task engagement modes—by round.

Task Engagement Mode	Comparison	N	Difference (Median)	Direction	V	Adj *p*	R	r_CI_lower	r_CI_upper
Passive	Round 1 vs. Round 2	14	−0.23	Decrease	62.5	0.21	−0.17	−0.73	0.36
Passive	Round 2 vs. Round 3	9	0.17	Increase	17.0	1.00	0.22	−0.42	0.89
Passive	Round 1 vs. Round 3	9	0.13	Increase	23.0	1.00	−0.02	−0.69	0.65
Active	Round 1 vs. Round 2	14	−0.05	Decrease	50.5	1.00	0.03	−0.50	0.53
Active	Round 2 vs. Round 3	9	0.00	No Change	14.0	1.00	0.34	−0.34	0.85
Active	Round 1 vs. Round 3	9	0.00	No Change	17.0	1.00	0.22	−0.49	0.77
Constructive/Interactive	Round 1 vs. Round 2	14	0.22	Increase	0.0	0.00	0.88	0.88	0.88
Constructive/Interactive	Round 2 vs. Round 3	9	0.00	No Change	13.5	1.00	0.36	−0.32	0.79
Constructive/Interactive	Round 1 vs. Round 3	9	0.25	Increase	7.5	0.32	0.59	0.02	0.85

**Table 7:** Frequency and percentage of instances of SRL promotion categories in the whole sample.

SRL Category	SRL Sub-Category	Round 1 (N = 14)		Round 2 (N = 14)		Round 3 (N = 9)	
		N	%	N	%	N	%
Forms of promotion	Explicit strategy	19	2.8	14	1.8	14	3
	Implicit strategy	208	30.1	236	29.6	146	31.3
	Knowledge/beliefs	42	6.1	23	2.9	23	4.9
	Metacognitive reflection	213	30.9	217	27.3	102	21.9
	Metacognitive support	208	30.1	306	38.4	181	38.8
Capabilities	Cognitive	407	59	338	42.5	194	41.6
	Metacognitive	149	21.6	223	28	107	23
	Motivational	90	13	177	22.2	119	25.5
	Affective	2	0.3	7	0.9	6	1.3
	Resource management	42	6.1	51	6.4	40	8.6
Domain	General	512	74.2	685	86.1	428	91.8
	Specific	178	25.8	111	13.9	38	8.2
Manner of promotion	Direct verbal	371	53.8	477	59.9	302	64.8
	Modelling	25	3.6	34	4.3	11	2.4
	Prompting	294	42.6	285	35.8	153	32.8
Benefit of use	Explanation: content	93	13.5	79	9.9	45	9.7
	Explanation: benefit	59	8.6	44	5.5	48	10.3
	Transfer	3	0.4	0	0	1	0.2
	None	535	77.5	673	84.5	372	79.8
**Total # instances**		690		796		466	

*Note:* For each of the SRL categories (“SRL category” column), there is one sub-category (“SRL sub-category” column) assigned for each utterance, meaning that the values in the percentage columns relate specifically to that SRL category.

**Table 8:** Wilcoxon signed-rank tests (Holm-adjusted) for SRL promotion utterances: comparing differences between rounds.

SRL Category	SRL Sub-Category	Comparison	N	Difference (Median)	Direction	V	Adj *p*	r	r_CI_Lower	r_CI_Upper
Forms of promotion	Explicit strategy	Round 1 vs. Round 2	14	0	No Change	27	0.72	0.00	−0.09	0.78
		Round 2 vs. Round 3	9	0.02	Increase	5	0.45	0.69	0.18	0.89
		Round 1 vs. Round 3	9	0.02	Increase	11	0.72	0.45	−0.14	0.89
	Implicit strategy	Round 1 vs. Round 2	14	−0.07	Decrease	62	1	−0.16	−0.68	0.36
		Round 2 vs. Round 3	9	0.09	Increase	14	1	0.34	−0.38	0.81
		Round 1 vs. Round 3	9	0	Increase	26	1	0.14	−0.65	0.57
	Knowledge/beliefs	Round 1 vs. Round 2	14	−0.01	Decrease	54	0.78	−0.03	−0.55	0.48
		Round 2 vs. Round 3	9	0.02	Increase	16	0.96	0.26	−0.45	0.85
		Round 1 vs. Round 3	9	0	No Change	19	0.96	0.00	−0.53	0.73
	Metacognitive Reflection	Round 1 vs. Round 2	14	−0.03	Decrease	55	0.9	−0.04	−0.56	0.46
		Round 2 vs. Round 3	9	−0.12	Decrease	41	0.09	−0.73	−0.89	−0.30
		Round 1 vs. Round 3	9	−0.09	Decrease	34	0.38	−0.45	−0.89	0.18
	Metacognitive Support	Round 1 vs. Round 2	14	0.06	Increase	26	0.2	0.45	−0.04	0.81
		Round 2 vs. Round 3	9	0	Increase	18	0.64	0.18	−0.49	0.89
		Round 1 vs. Round 3	9	0.09	Increase	4	0.09	0.73	0.30	0.89
Capabilities	Cognitive	Round 1 vs. Round 2	14	−0.25	Decrease	90	0.06	−0.63	−0.83	−0.13
		Round 2 vs. Round 3	9	0.12	Increase	16	0.48	0.26	−0.34	0.89
		Round 1 vs. Round 3	9	−0.23	Decrease	43	0.06	−0.81	−0.89	−0.53
	Metacognitive	Round 1 vs. Round 2	14	0.03	Increase	29	0.3	0.39	−0.11	0.78
		Round 2 vs. Round 3	9	−0.09	Decrease	38	0.24	−0.61	−0.89	−0.06
		Round 1 vs. Round 3	9	0.04	Increase	16	0.48	0.26	−0.38	0.85
	Motivational	Round 1 vs. Round 2	14	0.05	Increase	11	0.03	0.70	0.33	0.88
		Round 2 vs. Round 3	9	0.06	Increase	14	0.34	0.34	−0.34	0.85
		Round 1 vs. Round 3	9	0.13	Increase	5	0.08	0.69	0.18	0.89
	Resource management effective	Round 1 vs. Round 2	14	0.01	Increase	36	1	0.28	−0.26	0.71
		Round 2 vs. Round 3	9	−0.05	Decrease	25	1	−0.10	−0.77	0.53
		Round 1 vs. Round 3	9	0.08	Increase	18	1	0.18	−0.53	0.77
	Resource management effective	Round 1 vs. Round 2	14	0	No Change	2	0.54	0.00	0.71	0.88
		Round 2 vs. Round 3	9	0	No Change	7	1	0.00	0.02	0.89
		Round 1 vs. Round 3	9	0	No Change	0	0.54	0.00	0.89	0.89
Domain	General	Round 1 vs. Round 2	14	0.08	Increase	3	0.03	0.83	0.63	0.88
		Round 2 vs. Round 3	9	−0.01	Decrease	22	0.62	−0.02	−0.69	0.61
		Round 1 vs. Round 3	9	0.03	Increase	9	0.46	0.53	−0.06	0.89
	Specific	Round 1 vs. Round 2	14	−0.08	Decrease	52	0.03	−0.01	0.60	0.53
		Round 2 vs. Round 3	9	0.01	Increase	14	0.62	0.34	0.30	0.85
		Round 1 vs. Round 3	9	−0.03	Decrease	27	0.46	−0.18	0.77	0.49
Manner of promotion	Direct verbal	Round 1 vs. Round 2	14	0.08	Increase	33	0.69	0.33	−0.21	0.75
		Round 2 vs. Round 3	9	0	Increase	17	0.69	0.22	−0.49	0.77
		Round 1 vs. Round 3	9	0.17	Increase	12	0.69	0.42	−0.22	0.89
	Modelling	Round 1 vs. Round 2	14	0	No Change	23	1	0.00	−0.01	0.80
		Round 2 vs. Round 3	9	0	No Change	0	0.3	0.00	0.89	0.89
		Round 1 vs. Round 3	9	0	No Change	9	1	0.00	−0.10	0.89
	Prompting	Round 1 vs. Round 2	14	−0.07	Decrease	81	0.24	−0.48	−0.85	0.01
		Round 2 vs. Round 3	9	−0.01	Decrease	33	0.48	−0.42	−0.89	0.22
		Round 1 vs. Round 3	9	−0.17	Decrease	33	0.48	−0.42	−0.85	0.26
Benefit of use	Benefit of use	Round 1 vs. Round 2	14	0.02	Increase	57	1	0.08	−0.56	0.48
		Round 2 vs. Round 3	9	0.04	Increase	17	1	0.22	−0.49	0.77
		Round 1 vs. Round 3	9	0.04	Increase	23	1	0.02	−0.65	0.69
	None	Round 1 vs. Round 2	14	−0.02	Decrease	48	1	−0.08	−0.48	0.56
		Round 2 vs. Round 3	9	−0.04	Decrease	28	1	−0.22	−0.77	0.49
		Round 1 vs. Round 3	9	−0.04	Decrease	22	1	−0.02	−0.69	0.65

*Note:* Previous sub-categorizations of benefit of use have been combined (i.e., benefit of use vs. none).

## Data Availability

The original data presented in the study are openly available in OSF at https://osf.io/36fna/?view_only=ff5d71e0d4174110b5f7b782cf34aaef accessed on 29 October 2025.

## Abbreviations

The following abbreviations are used in this manuscript:

BALT	Beliefs about learning and teaching
COVID	Coronavirus disease
ICAP	Interactive, constructive, active, passive
ICAP-CG	Interactive, constructive, active, passive coding guide
IMPROVE	Introducing new concepts, metacognitive questioning, practicing, reviewing and reducing difficulties, obtaining mastery, verification, enrichment
PD	Professional development
PDP	Professional development programs
SRL	Self-regulated learning
SRL TPF	Self-regulated learning teacher promotion framework
SRT	Self-regulated teaching
STEM	Science, technology, engineering, mathematics
TIMMS	Third International Mathematics and Science Study
TSE	Teacher self-efficacy
